# Comparison of Oleogels Obtained by Emulsion Template Method Using Low Molecular Weight Hydroxypropyl Methylcellulose (HPMC) with Fish and Vegetable Oils

**DOI:** 10.3390/gels12040319

**Published:** 2026-04-08

**Authors:** Alonso Escobar, Leticia Montes, Amaya Franco-Uría, Ramón Moreira

**Affiliations:** Aquatic One Health Research Center (iARCUS), Department of Chemical Engineering, Universidade de Santiago de Compostela, Rúa Lope Gómez de Marzoa, s/n, 15782 Santiago de Compostela, Spain; alescobar2019@udec.cl (A.E.); leticia.montes.martinez@usc.es (L.M.); amaya.franco@usc.es (A.F.-U.)

**Keywords:** colour, oil binding capacity, oil rheology, texture, viscoelasticity

## Abstract

This work evaluated the influence of oil type (sunflower vs. fish oil) and hydroxypropyl methylcellulose (HPMC) concentration on the properties of oleogels obtained by the emulsion-templated method. Oil-in-water emulsions were prepared and air-dried to produce oleogels containing 2.9–5.8% (*w*/*w*) HPMC. All oleogels exhibited solid-like behaviour, with viscoelastic moduli increasing with polymer concentration, and showed a high thermal stability. At a comparable HPMC content, fish oil oleogels developed stiffer networks than those obtained with sunflower oil. Texture analysis indicated a linear increase in hardness with HPMC content across both oils, while cohesiveness and adhesiveness were more influenced by oil nature. Oil-binding capacity (OBC) increased markedly with polymer content, exceeding 90% in most systems. However, fish oil oleogels consistently showed lower retention. Colour parameters were only slightly affected by HPMC concentration and were mainly determined by the intrinsic colour of each oil. Overall, both oil type and polymer concentration were shown to be critical factors determining the structural, mechanical, and functional characteristics of HPMC-based oleogels, providing useful information for the development of structured lipid systems as potential substitutes for conventional solid fats.

## 1. Introduction

Cardiovascular disease remains the leading cause of mortality worldwide. One of the associated risk factors is the consumption of fats, particularly trans and saturated fats [1,2]. Their widespread use in processed foods, such as margarine, shortenings, and baked goods (cookies, biscuits, cakes), is due to their low cost and favourable physicochemical properties [1].

Trans and saturated fat intake should not exceed 1% and 10% of total caloric intake, respectively, with replacement by unsaturated lipids recommended to reduce the risks of heart disease, type 2 diabetes, and obesity [1,3]. Several countries, including Denmark, Lithuania, Poland, Saudi Arabia, and Thailand, have implemented regulations to eliminate trans fats from industrial food production [4]. The replacement of trans and saturated fats poses a significant challenge for the food industry, as they provide structural integrity and solid texture to diverse products [5]. A promising strategy is the structuring of oils into oleogels. These semi-solid systems reproduce the textural attributes of animal fats while being enriched in unsaturated lipids and containing reduced levels of saturated and trans fats [6]. For food applications, oleogels typically consist of more than 90% oil by mass [7]. This enables formulation from healthy oils (sunflower, canola, and fish) rich in polyunsaturated fatty acids with potential cardiovascular benefits. The successful development of oleogels requires a comprehensive understanding of the physicochemical properties of both the oils and the gelling agents. Accordingly, a wide range of preparation methods (direct, indirect, and semi-direct), gelling agents (e.g., fatty acids, waxes, xanthan gum, polysaccharides), and oils (e.g., sunflower, canola, olive, soybean, fish) have been investigated.

Most polymers used in the food industry are predominantly hydrophilic (such as native and many modified starches, proteins, gums, etc.), and therefore cannot structure oils directly [8]. Consequently, indirect methods have been developed, taking advantage of the diversity, availability, and physicochemical properties of food polymers. One of the most widely employed approaches is the emulsion template method. This involves preparing a stable oil-in-water (O/W) emulsion stabilized by a gelling agent, followed by the removal of water through drying methods such as convective, vacuum, or freeze drying [9]. The dried semi-solid material is then gently homogenized to obtain the oleogel [10].

Hydroxypropyl methylcellulose (HPMC), a cellulose-derived amphiphilic polymer substituted with hydroxypropyl and methoxyl groups, exhibits affinity for both water and nonpolar molecules [11]. Generally Recognized As Safe (GRAS) and widely used in pharmaceutical, cosmetic, and food industries, HPMC is cost-effective and associated with health benefits including reductions in cholesterol and postprandial glucose and insulin levels [12,13].

In recent decades, there has been increasing interest in incorporating fish oil into the human diet due to its high content of eicosapentaenoic acid (EPA) and docosahexaenoic acid (DHA). These omega-3 polyunsaturated fatty acids exhibit protective effects against cardiovascular disease, diabetes, asthma, arrhythmias, and atherosclerosis [14,15]. Nevertheless, intake of these compounds remains below recommended levels for many individuals [15]. Fish oil can be obtained from lipid-rich subproduct valorization of both fishing captures and aquaculture, increasing the opportunities of incorporating this oil into foods for human consumption.

Fish oil oleogels formulated with natural waxes have been previously studied by dissolving the waxes in the oil phase at elevated temperatures, followed by cooling to form the oleogel [16,17]. In contrast, the preparation of such oleogels using the emulsion template method remains unexplored. This study evaluates the rheological, textural, and physical properties of fish oil oleogels prepared with varying HPMC concentrations via the emulsion template method and compares them with the properties of sunflower oleogels prepared with the same method and structuring agent.

## 2. Results and Discussion

### 2.1. Rheology of Employed Vegetable and Fish Oils

**Figure 1:**
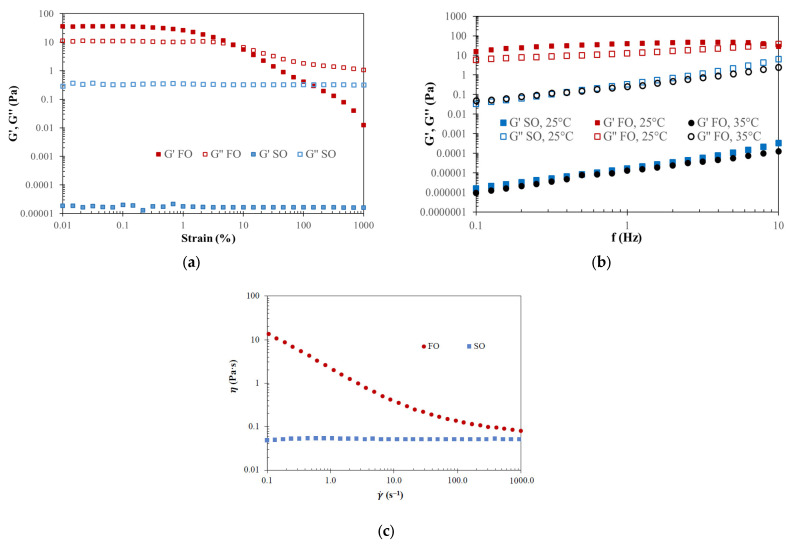
Strain at 25 °C (**a**), frequency at 25 and 35 °C (**b**) sweeps and flow curves at 25 °C (**c**) of vegetable (sunflower, SO) and fish (chub mackerel, FO) oils.

Figure 1 shows strain (a), frequency (b) sweeps and flow curves (c) of vegetable (sunflower, SO) and fish (chub mackerel, FO) oils. As expected, sunflower oil showed marked viscous behaviour (G″ > G′) in the LVR, but fish oil exhibited a more complex trend. In the strain sweep, at small strains (<0.3%, yield point), plateaus (LVR) for G′ and G″ were observed with a predominant elastic character. At higher strains, the G′ values decreased until they achieved a crossover point (flow point, at 8% strain), and beyond this point, viscous character was predominant. Considering these results, a common strain (0.1%) was selected to carry out the frequency sweep. The spectra of both tested oils were different. Sunflower oil showed a typical trend of a viscous liquid with G″ >> G′ (5 decades) with a pronounced dependence with frequency. Nevertheless, fish oil showed a flatter profile, with G′ > G″ revealing the presence of a gelled structure at low frequencies, achieving a gel point at high frequency (8.3 Hz). The different behaviour of both oils can be explained by the high saturated fatty acid content of fish oils with high melting points above 25 °C [3]. From the stationary curves (Figure 1c), sunflower oil can be considered as a Newtonian fluid with an average dynamic viscosity of 0.05 Pa·s, while fish oil had a pronounced shear-thinning behaviour, with the reduction in apparent viscosity from 15 to 0.09 Pa·s when increasing shear rate from 0.1 to 10^3^ s^–1^.

Temperature sweeps (Figures S1 and S2, Supplementary Information) of fish oil allow the determination of the melting point (damping factor, tan(δ) = G″/G′ = 1). This phase transition occurred at 32 ± 1 °C. Above this temperature, fish and sunflower oils could be classified rheologically in a similar way. In fact, Figure 1b shows that the frequency sweep of fish oil at 35 °C it is like those obtained for sunflower oil at 25 °C. Consequently, the respective temperatures were selected to maintain similar rheological conditions during the emulsion formation.

### 2.2. Rheology of Oleogels

**Figure 2:**
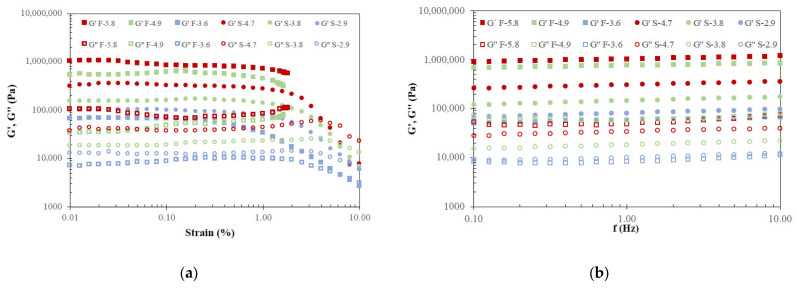
Strain (**a**) and frequency (**b**) sweeps of sunflower and fish oil oleogels at several HPMC contents.

Figure 2 shows the strain and frequency sweeps of sunflower and fish oil oleogels with different concentrations of HPMC. In the strain sweep inside the linear viscoelastic region (LVR), it was observed that G′ > G″ in all systems, indicating predominantly solid-like behaviour. As expected, regardless of the oil used in the oleogels, both moduli significantly increased with HPMC content, but at similar content (F-4.9 and S-4.7), fish oil oleogels showed higher values. Nevertheless, sunflower oil oleogels formulated at low HPMC content (S-2.9) showed higher values of both viscoelastic moduli than fish oil oleogels with higher HPMC content (S-3.8), indicating that the different nature of the employed oils is critical for the final oleogel features. In sunflower oil oleogels, the LVR slightly increased with increasing HPMC content, and the critical strain (the maximum strain at which the LVR is maintained) varied from 0.5 to 1.0% (Figure 2a). Fish oil oleogels prepared at intermediate and high HPMC content (specifically, F-5.8 and F-4.9) presented limitations in the evaluation of their rheological behaviour at strains above 1.0%, as the maximum torque of the equipment was reached. This result suggests that these samples are difficult to deform outside the linear range and maintain their gel structure. At low HPMC content, the critical strain of fish oil oleogel was determined at 0.3%. In this fish oil sample (F-3.6), a crossover point (G′ = G″) was determined at 2%, and oleogels formed with sunflower oil also presented this flow point, increasing from 3 to 8% by decreasing HPMC content. These results indicate that gel structural resistance increased with HPMC content, and is also promoted with the use of fish oil in the formulation. The results regarding the effect of HPMC content agree with previous findings of oleogels formulated with vegetable oils [12,18]. From these results, a strain of 0.1% was selected to carry out the frequency sweeps.

Frequency sweep tests (Figure 2b) confirmed that all systems exhibited solid-like behaviour (G′ > G″) with strong gel character given by the weak frequency dependence of the elastic modulus. Increasing HPMC concentration resulted in higher viscoelastic moduli, consistent with observations from the strain sweeps. Specifically, G′ (measured at 1 Hz) of fish oil oleogels ranged from 60.7 ± 2.2 up to 1052 ± 13 kPa, and of sunflower oil oleogels from 89.2 ± 3.4 to 312 ± 6 kPa with increasing HPMC content. The viscoelastic character (evaluated by means of damping factor at 1 Hz) decreased with HPMC content in fish oil oleogels from 0.14 ± 0.02 (F-3.6) to 0.08 ± 0.01 (F-5.8), and was invariant in sunflower oil oleogels (0.11 ± 0.01).

These findings agree with previous studies reporting similar rheological trends in HPMC-based oleogels, where these trends were attributed to the formation of stronger three-dimensional networks with increasing polymer content [19,20]. Notably, the moduli measured in fish oil oleogels were approximately two orders of magnitude greater than those reported by Cheng et al. [21] for fish oil oleogels prepared with 6–14% beeswax, highlighting the high stiffness of the HPMC-based systems. Nevertheless, the tested oleogels with intermediate and low HPMC content showed viscoelastic moduli values in the same range as commercial butters and spreadable blends at 20 °C [22]. To the best of our knowledge, no prior reports have addressed the rheological behaviour of fish oil oleogels produced via the emulsion-template method.

**Figure 3:**
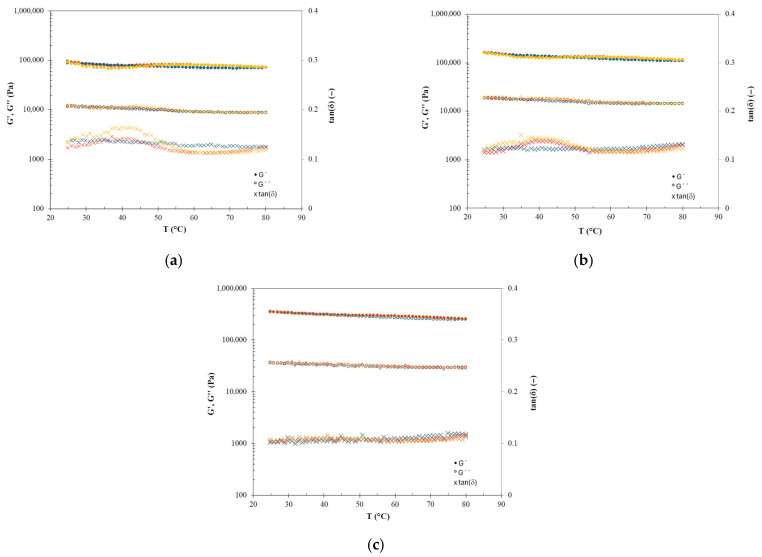
Temperature sweeps of sunflower oil oleogels with different HPMC content: (**a**) S-2.9, (**b**) S-3.8 and (**c**) S-4.7. Ramps: Red—First heating, blue—first and second cooling, and orange—second heating.

**Figure 4:**
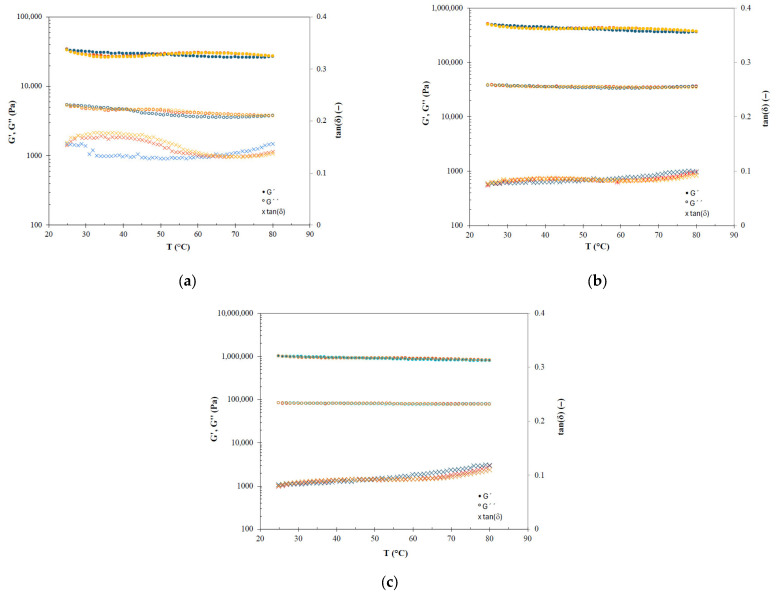
Temperature sweeps of fish oil oleogels with different HPMC content: (**a**) F-3.6, (**b**) F-4.9 and (**c**) F-5.8. Ramps: Red—First heating, blue—first and second cooling, and orange—second heating.

Figure 3 and Figure 4 show the thermal behaviour (25–80 °C) of oleogels prepared with sunflower and fish oil, respectively. The solid character (G′ > G″) always remained predominant in all tested oleogels, indicating that the gel structure is thermostable. Particularly, in the two cooling ramps, slight increases were observed in both modules as the temperature decreased, resulting in curves that were practically superimposed. However, during the heating ramps, alterations to this monotonous thermal behaviour were observed depending on the HPMC concentration at intermediate temperatures. Oleogels with high HPMC content (S-4.7 and F-5.8) showed the mentioned monotonous thermal behaviour during heating ramps with no significant differences regarding cooling ramps, meaning that these oleogels are highly thermostable.

As the HPMC concentration decreases, a decrease in the elastic modulus (a phenomenon that was clearer by the increase in the damping factor) was observed in the heating sweeps in the range from 30 to 50 °C. Specifically, in the sunflower oil oleogels, this fact was observed in oleogels S-3.8 and S-2.9 with a maximum damping factor around 40 °C. On the other hand, fish oil oleogels also showed the described trend, but it was very weak at intermediate content (F-4.9) and more evident at low HPMC concentrations (F-3.6) with damping factor peaks in the 35–40 °C interval. These phenomena must be related to the behaviour during the thermogelation of HPMC, in which at temperatures below the gelation temperature, there is a pronounced decrease in the elastic modulus [23,24]. These peaks disappeared with increasing HPMC content due to the high structural stability of the gel’s three-dimensional network. The high thermostability of oleogels prepared with HPMC (and other cellulose ethers) using different vegetable oils has already been reported along with their potential applications [12,20].

### 2.3. Texture Properties of Oleogels

Table 1 shows the textural properties obtained from the TPA test of sunflower and fish oil oleogels. Hardness of the sunflower oil and fish oil oleogels ranged from 4.4 to 9.6 N and from 1.1 to 9.5 N, respectively, and increased significantly (*p* < 0.05) with polymer concentration, in line with the increase in viscoelastic moduli observed in the frequency sweep of the oleogel. The increase in hardness was linear with HPMC content (R^2^ > 0.97) regardless of the type of oil. This behaviour is consistent with previous research on emulsion-template-structured oil oleogels, where increases in structuring agent concentration led to stronger networks, higher hardness, and greater mechanical strength due to enhanced polymer–polymer interactions during drying and matrix consolidation [18,19,25]. Fish oleogels were softer in comparison to vegetable oleogels at constant HPMC content. This could be explained by the presence of more polar compounds in higher unsaturated oil like fish oil, which could interact with gelators and reduce firmness [26,27]. According to Yang et al. [28], the hardness values of the oleogels were similar to those observed in commercially unsalted butters with high fat content (>75% *w*/*w*). Similarly, Ziarno et al. [29] reported that the hardness of some commercial butters ranged from 1.02 to 3.92 N, like the formulated oleogels in this work with intermediate and low HPMC content.

Adhesiveness showed values between 0.45 and 0.86 N·s for oleogels with sunflower oil, decreasing (*p* < 0.05) when a high concentration of HPMC was used. For oleogels with fish oil, adhesiveness ranged from 0.11 to 0.80 N·s and decreased significantly (*p* < 0.05) with increasing polymer concentration. Low variation in this parameter or opposite trends are, in general, observed in other works [19,30,31], although several factors (gelator type, drying method and temperature, or water content) can affect this property besides gelator concentration. Lower adhesiveness was noted in the fish oil oleogels at the common range of HPMC content. This may be due to part of the oil being released to the outer layer of the oleogel during compression. Adhesiveness values of the tested oleogels were lower than those observed in some commercial butters [28,29], but in the same range as those obtained in butter substitutes [29].

Cohesiveness for sunflower oleogels displayed the opposite behaviour to adhesiveness, increasing significantly only at the highest HPMC concentration. On the other hand, the cohesiveness of fish oil oleogels varies within a narrow range (from 0.30 to 0.36), in the same range as low-fat margarines and higher than commercial butter [32], without significant differences related to polymer concentration (*p* = 0.463). The limited increase in cohesiveness is consistent with other works showing that oils with high PUFA content—such as flaxseed oil or fish oil—often yield weaker, less cohesive oleogels due to poorer structuring capacity and reduced compatibility with cellulose ethers [26]. For the S-3.8 and F-3.6 samples (with the most comparable concentrations among oils), the cohesiveness of the fish oil oleogel proved to be slightly higher than that of the sunflower oil oleogel. However, upon increasing the polymer concentration, no significant variation in this property for fish oil oleogels was found, while there was a significant increase for sunflower oil.

Elasticity in sunflower oleogels increased (*p* < 0.05) from 63 to 68% for S-2.9 and S-3.8. However, this parameter decreased to 59% when HPMC concentration increased (S-4.7). Elasticity can increase with polymer concentration until excessively rigid networks form at high gelator levels. Previous works reported an elasticity decrease with increasing gelator concentration and hardness [19,33]. On the other hand, elasticity increased significantly from 60 to around 73% at high HPMC content in fish oil oleogels. As previously mentioned, this behaviour may be attributed to the greater presence of gelator-interacting compounds in highly polyunsaturated oils, which can yield networks that, while less cohesive and structurally weaker, remain more deformable when sufficient polymer is present.

### 2.4. Oil Binding Capacity (OBC)

**Figure 5:**
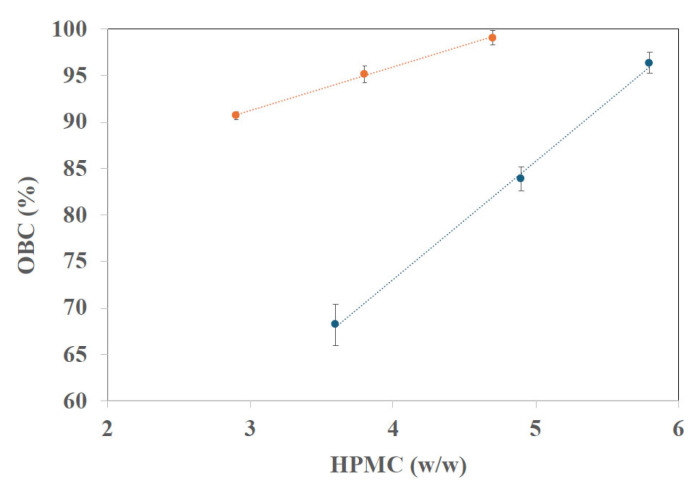
Oil binding capacity (OBC) of oleogels vs. HPMC concentrations and linear adjustment. Orange: sunflower oil oleogels; blue: fish oil oleogels.

Figure 5 illustrates the oil-binding capacity (OBC) of sunflower oil and fish oil oleogels structured with HPMC. For sunflower oleogels, the OBC increased significantly (*p* < 0.05) from 90.7 up to 99.1% with increasing HPMC content. These results are consistent with those reported by Saavedra et al. [34], where similar OBC values were obtained in oleogels prepared from emulsions containing 2 and 3% HPMC.

In fish oil oleogels, OBC also increased significantly (*p* < 0.05) with polymer content, ranging from 68.2 to 96.4%. Considering an OBC threshold of 80% as an indicator of good oil retention, F-3.6 oleogels did not reach this value. It can be observed in Figure 5 that the fish oil oleogels showed lower oil retention than the sunflower oil oleogels at the same range of HPMC content. Moreover, increasing HPMC concentration led to a linear increase (R^2^ > 0.99) in OBC for both oleogels made with different types of oil. Overall, the related literature indicates that increasing the concentration of the structuring agent, regardless of the method and oil employed, leads to higher oil retention indices [25,31,35,36,37]. This fact was also observed in previous reports on oleogels structured with HPMC as the gelling agent [19,20,34]. In line with these results, a linear relationship could be established with oleogel hardness (which also significantly increased with HPMC content) and OBC, regardless of the oil employed in oleogel formation (Figure S3). This result was also consistent with the lower oil retention obtained in the fish oil oleogels compared to those made with sunflower oil. The steeper slope of the linear fit, in Figure 5, for the OBC of fish oil oleogel indicated that it is necessary to increase the HPMC concentration in this oleogel to achieve OBC values similar to those of the sunflower oil oleogels, in accordance with the reported interactions of PUFA oil with HPMC, which may hinder matrix structuring [26]. On the other hand, the OBC values for fish oil oleogels at the maximum concentration of HPMC tested were close to those reported by other authors [16,21] for fish oil oleogels structured with different waxes.

### 2.5. Colour Features of Oleogels

Table 2 shows the colorimetric parameters of the sunflower and fish oil oleogels for the different HPMC concentrations tested. For the sunflower oil oleogels, lightness (L*) varied within a narrow range (between 41.5 and 43.3), with no significant differences (*p* = 0.120) across the evaluated polymer concentrations. These results agreed with the reported data for other templated oleogels from olive oil using different content of chitosan (0.7 and 0.8% *w*/*w*) as a structuring agent [38]. The chroma (C*) of the sunflower oil oleogels ranged between 5.9 and 6.3 and, as with L*, no significant differences were found among the samples (*p* = 0.335). Likewise, no significant differences (*p* = 0.438) were observed for the hue angle (h*) with polymer concentration. The h* values ranged from 101° to 103°, indicating a yellowish coloration.

In the fish oil oleogels, L* varied from 27.8 to 32.8, with a significant increase observed between the lowest HPMC content and the higher ones. The chroma of the oleogels ranged between 18.7 and 20.2, decreasing with increasing HPMC concentration, with significant differences (*p* < 0.05) relative to the oleogel with the lowest concentration. Lastly, the hue angle increased significantly (*p* < 0.05) with polymer concentration, from 72.9° to 76.7°, indicating that the oleogels exhibited an orange coloration like fish oil.

The lightness of the sunflower oil oleogels was higher than that of the fish oil oleogels at all the tested HPMC concentrations, which was expected given that sunflower oil is refined and naturally lighter than fish oil. The C* values of the fish oil oleogels were higher than those of the sunflower oil oleogels, whereas the h* was lower. Although significant differences were detected for some samples, particularly for fish oil oleogels, the HPMC content slightly modified the colourimetric parameters within each type of oil, meaning that colour differences in the oleogels were mainly attributable to the intrinsic color of the encapsulated oil, as reported in other studies [39,40].

## 3. Conclusions

Oleogels structured with HPMC showed predominantly elastic behaviour (G′ > G″), and both elastic and viscous moduli increased with increasing polymer concentration. At comparable HPMC levels, fish oil oleogels developed more rigid structures than those formulated with sunflower oil, confirming the strong influence of oil nature on network formation. All oleogels presented high thermostability, but at low and intermediate HPMC content, a decrease in elastic modulus, related to HPMC thermogelation, between 30 and 50 °C was observed for heating ramps, whereas samples with higher polymer content remained unaffected throughout the temperature range. Textural properties were mainly governed by HPMC content. Hardness increased linearly with polymer content regardless of the oil employed. Adhesiveness and cohesiveness were more dependent on the characteristics of the oils, with fish oil oleogels displaying lower adhesiveness and restricted cohesiveness due to the higher unsaturation degree of the oil. Oil-binding capacity increased significantly with HPMC concentration for fish and vegetable oil oleogels, reaching values above 90% in most formulations. Fish oil oleogels showed lower OBC values than sunflower oil gels, and the formulation with the lowest HPMC content did not exceed the 80% retention threshold commonly considered satisfactory. Colour parameters were only slightly influenced by HPMC content and were mainly determined by the intrinsic colour of the oils. Fish oil oleogels were darker and exhibited higher chroma values than sunflower oil oleogels due to the natural pigmentation of the oil. These results allow us to conclude that fish oil with good nutritional and health characteristics can be employed to manufacture oleogels using the template method with satisfactory attributes in comparison to vegetable oil oleogels and common commercial fats.

## 4. Materials and Methods

### 4.1. Materials

Fish oil obtained from the skin and bones of Japanese mackerel (*Scomber japonicus*) was provided by the Marine Research Institute (IIM-CSIC). Sunflower oil was acquired in a local market. Hydroxypropyl methylcellulose (HPMC) with low molecular weight was employed with a viscosity of 80–120 cP in a 2% (*w*/*v*) aqueous solution at 20 °C and with methoxy and hydroxypropyl groups at proportions of 28.7% and 9.1%, respectively.

### 4.2. Preparation of Emulsions

Oil-in-water emulsions were prepared at several concentrations of HPMC (1.5, 2.0, and 2.5% *w*/*w*). All emulsions (100 g) were prepared at the highest oil-to-water ratio able to produce oleogels avoiding phase separation during emulsion preparation or during the drying step. This aspect is relevant because the use of the minimum water fraction reduces the energy consumption during drying for oleogel production. Fish and sunflower oleogels were able to be developed with maximum oil-to-water ratios of 40:60 and 50:50 (*w*/*w*), respectively.

Emulsions with sunflower oil were prepared according to the procedure described by Saavedra et al. [34]. HPMC was first dispersed in the oil phase using a four-blade stirrer (IKA-WERK RW 20 DZM, Staufen, Germany) at 280 rpm for 7 min. Cold water (~10 °C) was then gradually added over 1 min while stirring, allowing effective polymer hydration. The mixture temperature was maintained at 25 °C using a controlled water bath during homogenization using a high-energy dispersion unit (Ultraturrax T-25 Basic, IKA-WERK, Staufen, Germany) at 9500 rpm for 15 s, followed by a second cycle at 21,500 rpm for 40 s. During homogenization, the mixture was simultaneously stirred using an orbital shaker (Rotaterm, Selecta, Barcelona, Spain) at 120 rpm. Emulsions with fish oil were prepared following the same protocol, but homogenization was performed at 35 °C to decrease the oil viscosity and to approximate the rheology of fish oil to sunflower oil. Finally, the emulsions were allowed to stand at room temperature for 20 min to stabilize its microstructure before further analysis.

### 4.3. Formation of Oleogel

After the resting period, emulsions were convective air dried to yield the oleogel. Emulsions were spread in Petri dishes (11.5 cm diameter) at 0.20 cm thickness (18 g/dish). Drying was performed in a convective air dryer (Angelantoni Challenge 250, Massa Martana, Italy) at 80 °C, 10% relative humidity, and 2 m/s air velocity until the moisture content was less than 0.01 g H_2_O/g dry solids.

The resulting dried solids were homogenized at 6500 rpm until uniform paste formation with no uncrushed particles, yielding final HPMC concentrations of 3.6, 4.9, and 5.8% (*w*/*w*) in the case of fish oil oleogels and 2.9, 3.8, and 4.7% (*w*/*w*) for sunflower oil oleogels. Samples were then transferred to plastic ice cube trays and stored at 2 °C until analysis.

### 4.4. Rheological Characterization

Rheological characterization of the tested oils and oleogels was performed using a stress-controlled rheometer (MCR 301, Anton Paar GmbH, Graz, Austria) equipped with a 50 mm plate-plate geometry and a 1.5 mm gap for all measurements. Strain sweep tests were conducted at deformations ranging from 0.01% to 1000% for oils and emulsions and 0.01% to 10% for oleogels at a constant frequency of 1 Hz to determine the linear viscoelastic region (LVR). Frequency sweep measurements were performed between 0.1 and 10 Hz at 0.1% (oils and emulsions) and 0.01% (oleogels) fixed strain within the LVR. All experiments were carried out at 25 °C.

Temperature ramp tests were performed in the interval from 25 °C to 80 °C at a heating/cooling rate of 3 °C/min, comprising two heating ramps and two (intermediate and final) cooling ramps to evaluate the thermoreversible behaviour of the oleogels. The applied strain and frequency during these thermal sweeps were 0.01% and 1 Hz, respectively.

### 4.5. Texture Properties

Cylindrical oleogel samples with a diameter of 19.5 mm were compressed in a texturometer (TA.XT Plus, Stable Micro System, Surrey, UK) equipped with a cylindrical probe with a diameter of 25 mm (SMS P/S25). The samples were subjected to texture profile analysis (TPA) at 25 °C, which consisted of two consecutive compressions to 50% of the initial height at a descent speed of 1 mm/s and an applied threshold force of 0.1 N. The maximum hardness (N), adhesiveness (N·s), cohesiveness (—), and elasticity (%) of the compressed samples were obtained. Four replicate samples were analyzed for each batch of oleogel.

### 4.6. Oil Binding Capacity

Oil retention capacity was determined according to Lama et al. [38]. Oleogel samples (1 g, *n* = 4) were placed in pre-weighed Eppendorf tubes and centrifuged (HWLAB, HW12, Shiley, NW, USA) at 13,500 rpm (∼15,000× *g*) for 25 min. After centrifugation, supernatant oil was carefully removed and the Eppendorf tubes with the remaining oleogel were reweighed. OBC (%) was calculated according to Equation (1):

(1) $$OBC\;\left(\%\right)=\left(\frac{m_{2}-m_{t}}{m_{1}-m_{t}}\right)\cdot 100$$

where $m_{1}$ and $m_{2}$ are the weight (g) of the sample and tube before and after centrifugation, and $m_{t}$ is the weight (g) of the empty Eppendorf tube.

### 4.7. Colour Features

The colour of the oleogels (*n* = 4) was measured using a colorimeter (Konica Minolta CR-400, Osaka, Japan), previously calibrated using a calibration plate. The parameters defined within the CIELAB space (L*, a*, b*) were obtained. The chroma (C*) and hue angle (h*) were evaluated by means of Equation (2) and Equation (3), respectively [41].

(2) $$C^{*}\;\left(-\right)=\sqrt{{\left(a^{*}\right)}^{2}+{\left(b^{*}\right)}^{2}}$$

(3) $$h^{*}\;\left({}^{\circ}\right)={tan}^{-1}\left(\frac{b^{*}}{a^{*}}\right)\;$$

### 4.8. Statistical Analysis

Statistical analyses were carried out using IBM SPSS Statistics 29 software (SPSS 29, Chicago, IL, USA). Analysis of variance (ANOVA) was performed employing the general linear model at a 95% confidence level. HPMC concentration for each polymer type was considered the independent variable, while the dependent variables comprised the textural properties, oil-binding capacity (OBC), and colour parameters.

## Figures and Tables

**Table 1:** Textural properties of sunflower and fish oil oleogels at different HPMC concentrations. Capital (A, B, and C) and lowercase letters (a, b, and c) indicate statistically significant differences (*p* < 0.05) for sunflower and fish oil with gelator concentrations, respectively. Both cases were based on Duncan’s test with comparisons made independently.

Oleogel	Hardness (N)	Adhesiveness (N·s)	Cohesiveness (−)	Elasticity (%)
S-2.9	4.42 ± 0.37 ^C^	0.86 ± 0.07 ^A^	0.28 ± 0.03 ^B^	63.18 ± 4.32 ^A^
S-3.8	7.46 ± 0.57 ^B^	0.92 ± 0.06 ^A^	0.27 ± 0.01 ^B^	68.44 ± 4.23 ^A^
S-4.7	9.56 ± 0.66 ^A^	0.45 ± 0.10 ^B^	0.40 ± 0.03 ^A^	58.91 ± 1.96 ^B^
F-3.6	1.10 ± 0.26 ^c^	0.80 ± 0.14 ^a^	0.30 ± 0.06 ^a^	60.15 ± 6.58 ^b^
F-4.9	4.76 ± 0.52 ^b^	0.24 ± 0.07 ^b^	0.36 ± 0.05 ^a^	72.83 ± 4.87 ^a^
F-5.8	9.46 ± 0.56 ^a^	0.11 ± 0.07 ^b^	0.36 ± 0.05 ^a^	73.47 ± 6.91 ^a^

**Table 2:** Colour parameters of sunflower and fish oil oleogels prepared with HPMC. Letters (a and b) indicate statistically significant differences (*p* < 0.05) for fish oil with gelator concentrations. No significant differences for sunflower oil were found. Both cases were based on Duncan’s test with comparisons made independently.

Oleogel	L* (−)	C* (–)	h* (°)
S-2.9	43.31 ± 1.62	6.28 ± 0.48	103.05 ± 2.60
S-3.8	41.52 ± 1.00	5.98 ± 0.10	102.11 ± 0.55
S-4.7	42.96 ± 1.53	6.12 ± 0.47	101.73 ± 2.74
F-3.6	27.79 ± 0.40 ^b^	20.20 ± 0.35 ^a^	74.06 ± 1.29 ^b^
F-4.9	32.82 ± 0.68 ^a^	18.69 ± 0.43 ^b^	72.92 ± 0.43 ^b^
F-5.8	32.47 ± 0.21 ^a^	18.79 ± 0.67 ^b^	76.73 ± 0.76 ^a^

## Data Availability

The raw data supporting the conclusions of this article will be made available on request from the corresponding author.

## Supplementary Materials

The following supporting information can be downloaded at: https://www.mdpi.com/article/10.3390/gels12040319/s1. Figure S1: Temperature sweeps of fish (chub mackerel) oil. Trends of elastic (G′) and viscous (G″) moduli. Figure S2: Temperature sweeps of fish (chub mackerel) oil. Trend of damping factor (tan(d)). Figure S3: Linear relationship between hardness and Oil Binding Capacity (OBC) of tested oleogels.
