# Supplementary material for: Comparison of Oleogels Obtained by Emulsion Template Method Using Low Molecular Weight Hydroxypropyl Methylcellulose (HPMC) with Fish and Vegetable Oils
Source: Gels. 2026 Apr 8;12(4):319. doi: 10.3390/gels12040319 (PMC13115469; doi:10.3390/gels12040319)
Supplement: Supplementary file 1 [file gels-12-00319-s001.zip › gels-4223750-supplementary.pdf]

Supplementary Information

Comparison of oleogels obtained by emulsion template using low molecular weight hydroxypropyl methylcellulose (HPMC) with fish and vegetable oils

Authors: A. Escobar, L. Montes, A. Franco-Uría, R. Moreira

1. In Figures S1 and S2, temperature sweeps of fish oil are shown.

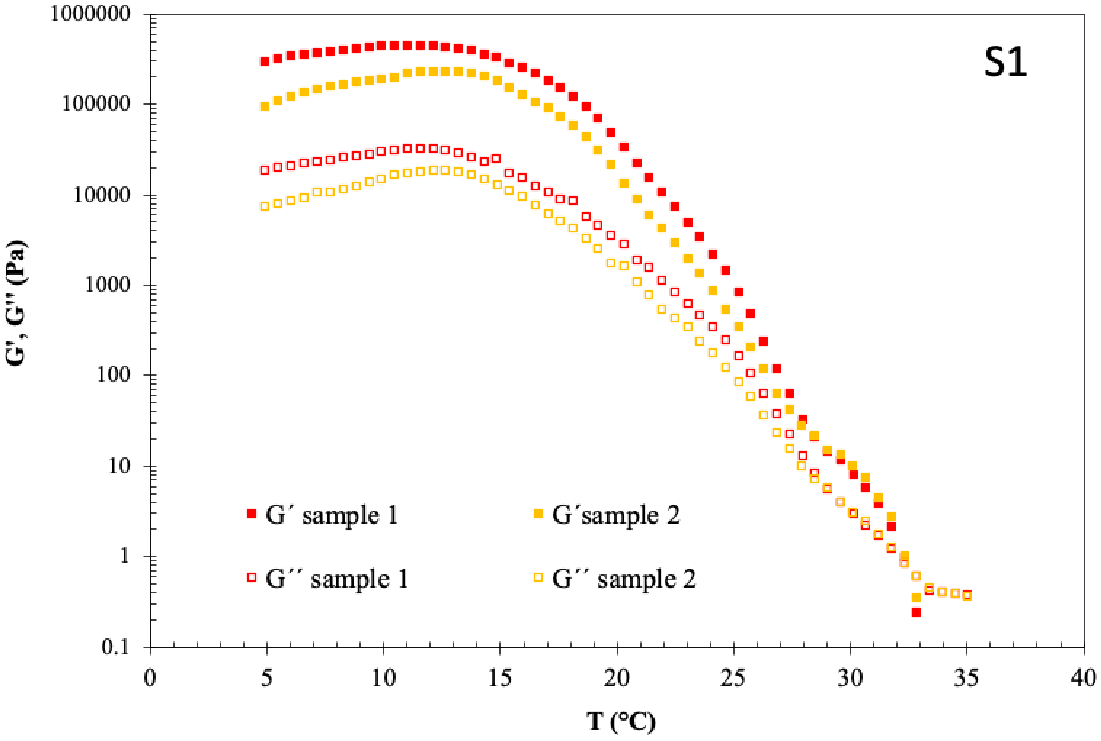

Figure S1. Temperature sweeps of fish (chub mackerel) oil. Trends of elastic (G′) and viscous (G′′) moduli.

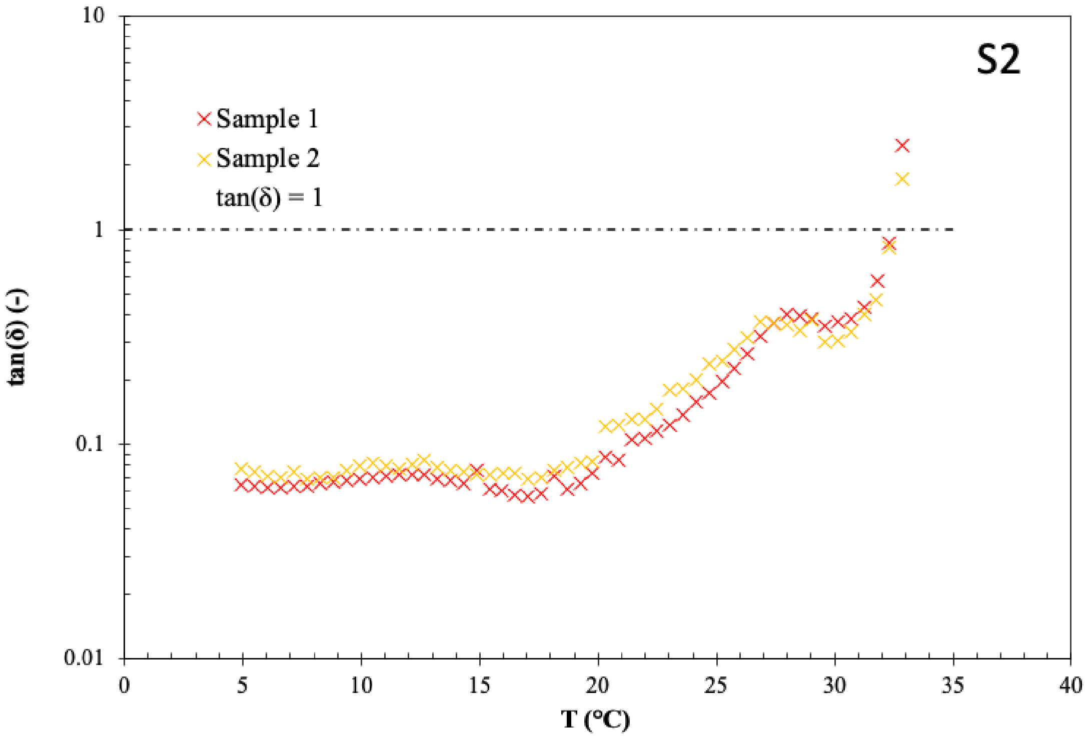

Figure S2. Figure S2. Temperature sweeps of fish (chub mackerel) oil. Trend of damping factor (tan(d))

2. Oil binding capacity *versus* hardness

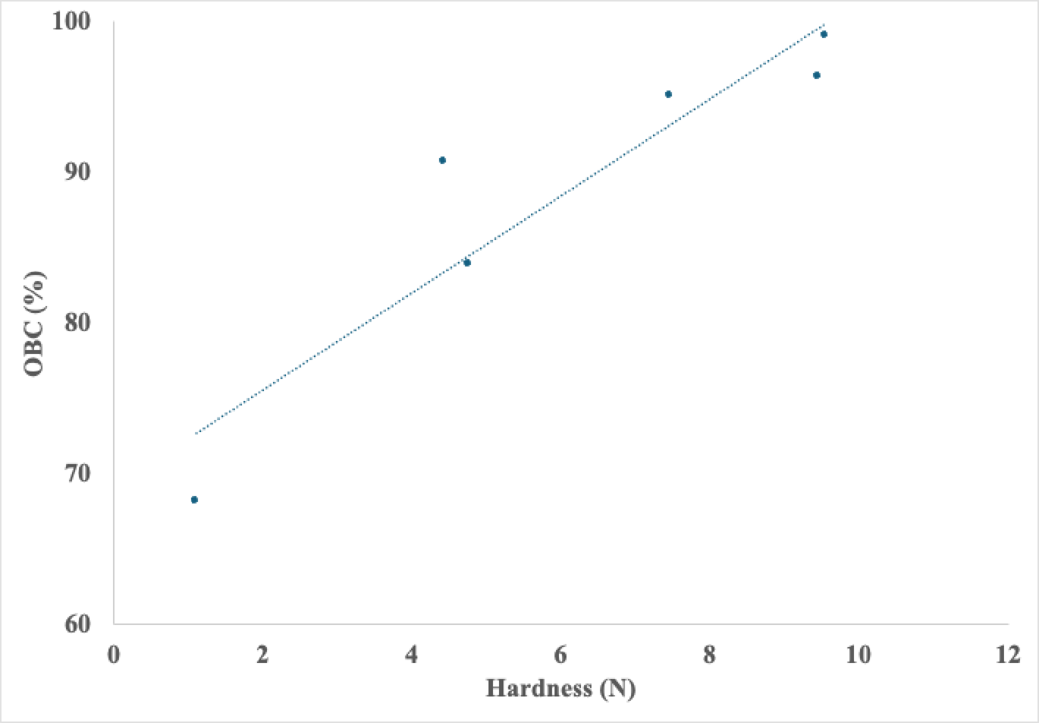

Figure S3: Linear relationship between hardness and Oil Binding Capacity (OBC) of tested oleogels
